# Treatment of the Clinical Symptoms of Osteoarthritis in the Elbow Joints of Dogs Using Nuclear Magnetic Resonance Therapy: A Randomized, Double-Blinded Trial

**DOI:** 10.3389/fvets.2020.500278

**Published:** 2020-11-13

**Authors:** Nikolaus Huels, Oliver Harms, Dana Keim, Karl Rohn, Michael Fehr

**Affiliations:** ^1^Clinic for Small Animals, University of Veterinary Medicine Hannover Foundation, Hanover, Germany; ^2^Institute for Biometry, Epidemiology and Information Processing, University of Veterinary Medicine, Hanover, Germany

**Keywords:** cubarthrosis, dog, elbow joint, gait analysis, arthrosis, osteoarthritis, nuclear magnetic resonance therapy

## Abstract

**Objectives:** To evaluate the effects of nuclear magnetic resonance therapy (MBST®) on the clinical symptoms of osteoarthritis (OA) in the elbow joints of dogs.

**Methods:** In this double-blind study, 28 dogs with lameness caused by OA in the elbow joint were randomly allocated to two groups: 14 dogs received nuclear magnetic resonance (NMR) therapy [treatment group (TG)], and 14 dogs received a placebo [placebo group (PG)] over a period of 7 consecutive days. Visual and objective gait analyses were performed before treatment (M1) and at 3 (M2) and 6 months (M3) after treatment. At M2 and M3 Symmetry indices (SI) of the peak vertical force (PVFz) and the vertical impulse (IFz), lameness scores, and pain scores were compared with their values at M1 to calculate the overall treatment effectiveness (OTE) score. We also documented additional pain medication and medical physiotherapy during the time of study. Finally, we measured the range of motion (ROM) in order to evaluate the functional development of the joint.

**Results:** The median OTE score of dogs in the TG indicates no change after 3 month and was improved after 6 months of treatment. There was an improvement of the median OTE score of dogs in the PG after 3 months of treatment. Further, the OTE scores of dogs in the PG were actually worse after 6 months.

Nevertheless, there were no significance differences in SIPVFz, SIIFz, ROM, and lameness- and pain scores between the TG and PG at M1, M2, and M3. When considering all collected parameters (excluding the ROM) to calculate the OTE, no significant difference between groups was measurable for the OTE.

**Conclusion:** There was a positive effect of NMR therapy (MBST®) on the treatment of OA in dogs. However, future studies should investigate the mechanisms underlying NMR therapy and the pathophysiology of OA to provide optimal treatments for patients.

**Clinical Significance:** Our results demonstrated that the response to NMR treatment was individualized for each dog. As an integral way of treating dogs with chronic OA, NMR therapy may be an alternative therapeutic approach to support traditional medications.

## Introduction

**Figure 1:**
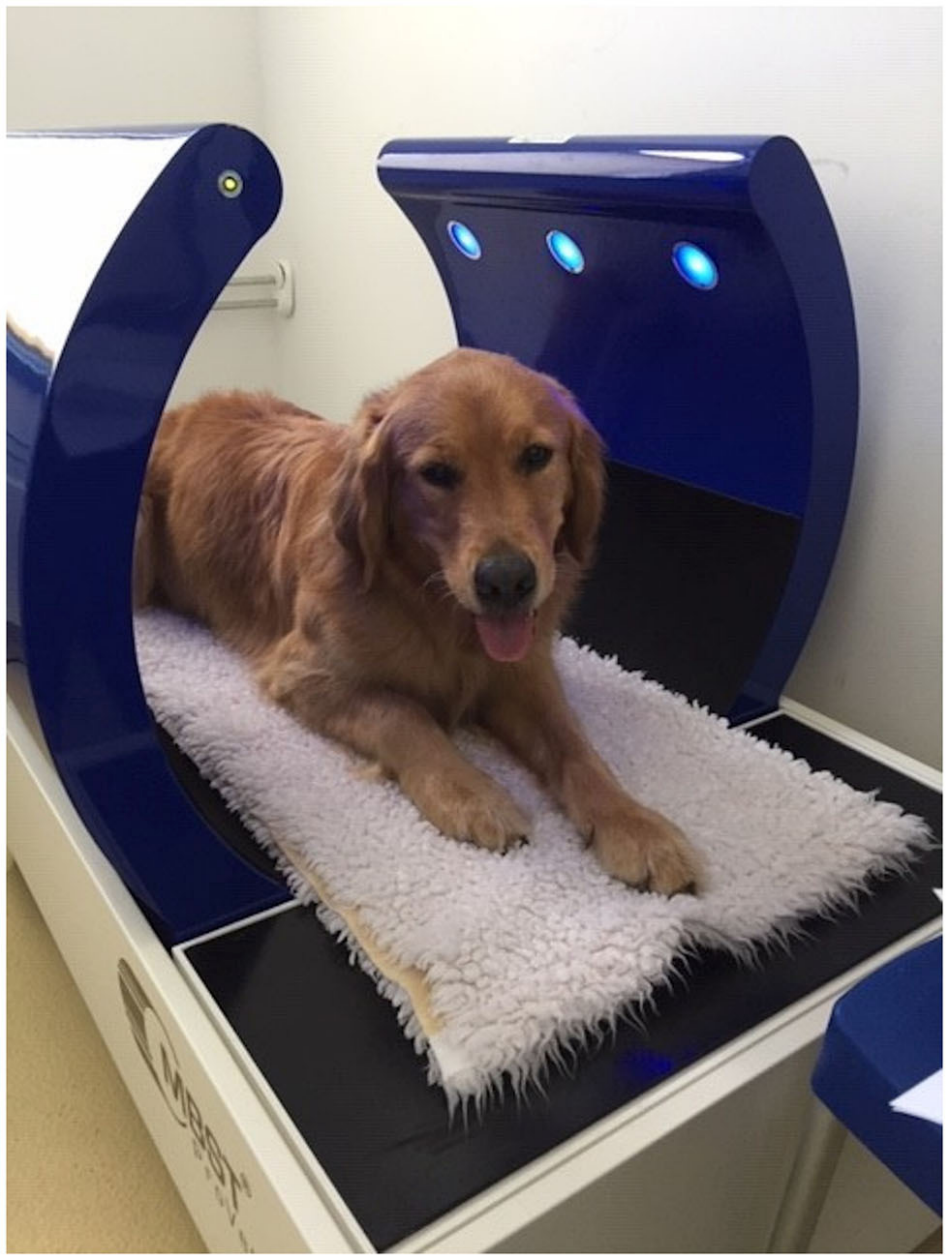
Picture of a dog in the MBST® Pro-Vet station. The NMR field is between the blue arches. The elbow should be within this area for the duration of treatment. NMR, nuclear magnetic resonance field.

Osteoarthritis (OA) is commonly observed in veterinary practice. Approximately 15% of all dogs in Germany suffer from a form of OA, and these animals require therapy (1, 2). OA results in pain and a loss of function of the affected joint, being associated with a considerable reduction in the quality of life for the dog and their owners. Additionally, studies have reported that chronic pain results in behavioral changes (3). Currently, the main goal of OA treatment is to reduce pain, lameness, and the progression of OA as well as to maintain the function of the joint (4, 5). Aside from traditional medical treatments, the attending veterinarian can also recommend various therapies, including joint injections, physiotherapy, and nutraceutical agents (6). Furthermore, a relatively new method, known as nuclear magnetic resonance (NMR) therapy, has become available in veterinary medicine. The so-called Molecular Biostimmulation (MBST® Figure 1) uses a 0.4–2.35 mT magnetic field that is combined with an interfering radio frequency signal (RFS). Similar to a magnetic resonance imaging system, the hydrogen protons align their magnetic moment in the direction of the magnetic field in NMR. When the RFS is applied, hydrogen protons change their direction and absorb energy. When the RFS is turned off again they relax back into the direction of the magnetic field and emit energy. On the recommendation of the manufacturer, a radiofrequency of 16 kHz was used for the optimal stimulation of the hydrogen protons in the articular surface.

*In vitro* studies revealed that NMR had a positive effect on cell proliferation (7). Moreover, Kullich et al. (8) used NMR in addition to a standardized physiotherapy protocol and reported that humans who were suffering from back pain experienced a significant reduction in pain after 3 months of NMR therapy (8). Further, Krpan and Kullich (9) suggested that NMR may reduce the risk of fractures in humans with osteoporosis, and NMR was able to reduce the amount of osteophytic proliferation in experimentally induced gonarthrosis in rabbits (10). Mucha et al. (11) conducted a double-blinded, prospective study that included dogs with radiographically confirmed OA in several joints. Although there were no significant differences between TG and PG, dogs in the TG experienced a significant improvement in lameness scores 3 months after NMR therapy. However, there were no measurable differences in lameness scores 6 months after therapy.

The aim of the present study was to investigate the effects of MBST® on dogs with OA in the elbow joint 3 and 6 months after treatment. We hypothesized that MBST® can significant improve the symptoms caused by osteoarthritis and be beneficial in the treatment of OA.

## Materials and Methods

### Study Population

**Figure 2:**
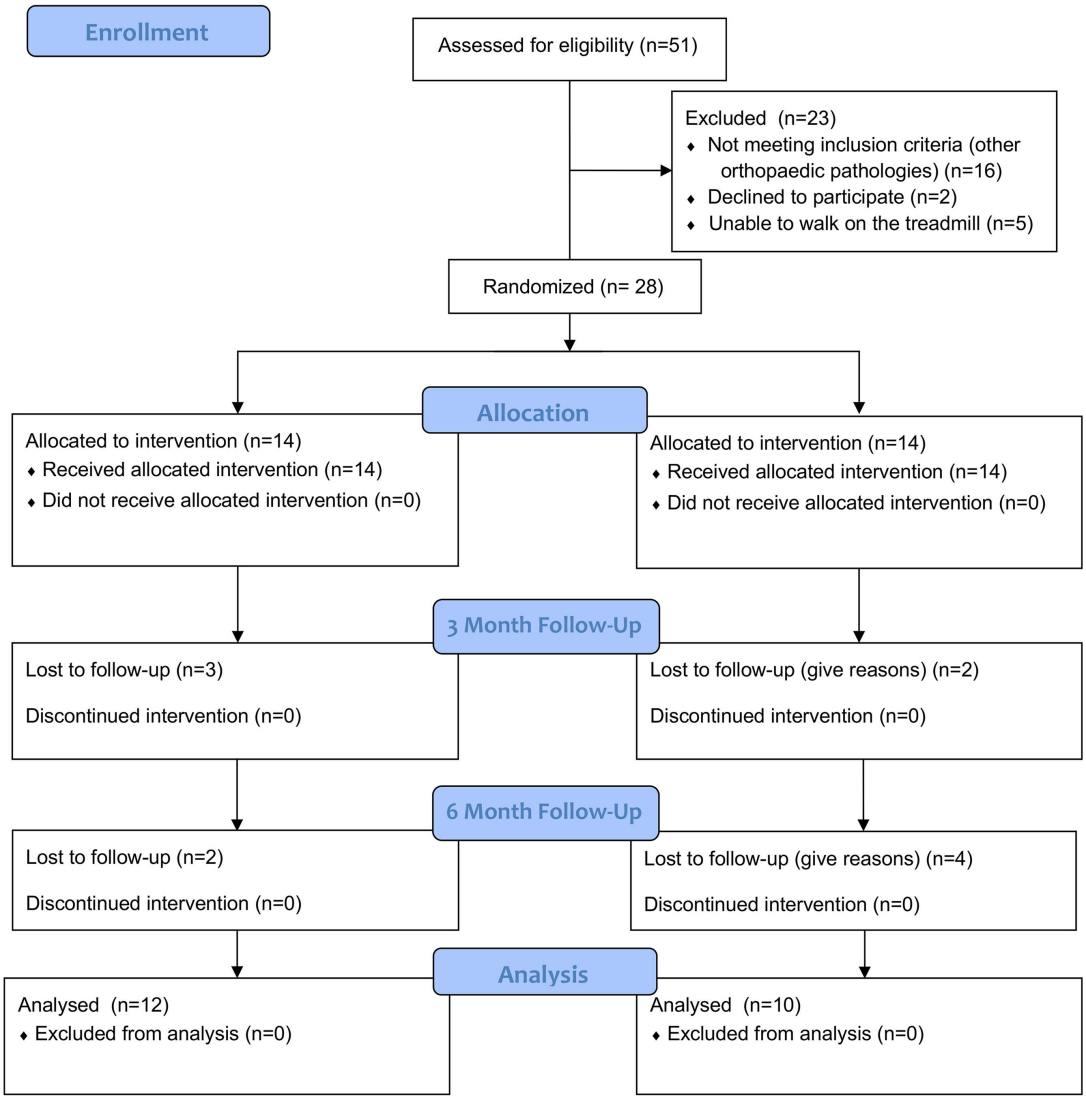
The consort flow diagram is created by NH. The data contained in the diagram was collected by NH.

A double-blinded, randomized clinical trial was performed. A total of 28 dogs with OA in the cubital joint were prospectively enrolled in the study, taken from the Clinic for Small Animal Medicine, University of Veterinary Medicine Hannover Foundation, Hannover, Germany from April 2018 to May 2019 (Figure 2). All dogs were presented with thoracic limb lameness and underwent a full clinical and orthopedic examination. If the lameness was not clearly attributable to the elbow joint, the dogs were excluded from the study. In order to verify the diagnosis of OA in the elbow joint and to exclude dogs with concurrent orthopedic pathologies, dogs were prospectively enrolled in the study if two radiographs per elbow and one lateral radiograph per shoulder were available. All dogs showed signs of pain during the palpation of the elbow of the affected limb. Dogs with bilateral OA were also included in the study if the lameness was clearly associated with one limb and cubital joint. Additionally, dogs were required to walk on a treadmill so that we could perform objective gait analysis. Similar to previous studies (11, 12), dogs receiving additional medical pain therapy or regular physiotherapy were also eligible to participate in the trial. However, there should not have been changes in medication or physiotherapy protocol for at least 4 weeks prior to the first measurement. Dogs with anxious or aggressive behavior were excluded from the study. All owners have agreed in writing to participate in the study.

### Randomization

Randomization was performed in accordance with a previous study (11). A special smart card was required to start the MBST®-Pro-Vet station prior to treatment administration. The manufacturer (MedTec Medical GmbH, Wetzlar, Germany) programmed 14 cards for the treatment group (TG) and 14 cards for the placebo group (PG), numbering them randomly from 1 to 28. In order to their entry number into the study the dogs were dedicated to the number of the smart card. Study investigators, academic supervisors and patient owners were blinded to treatment allocation.

### Nuclear Magnetic Resonance Therapy (MBST®)

We used a MBST®-Pro-Vet Station (MedTec Medical GmbH, Wetzlar, Germany) for NMR therapy in the current study (Figure 1). The static background field had a magnitude of 0.28 mT and a RFS of 16 kHz in the TG. The smart card contained all of the information that was needed for the three-stage OA program, which was performed on the following 7 consecutive days, including the weekend. Additionally, it was impossible to differentiate between the treatment and placebo groups. During the 1 h visit, the animal was placed in the area of the NMR field in the presence of the owner. Although the dogs were able to move during treatment, they were required to remain within the NMR field. Sedation was not necessary. According to the operating instructions, therapy should have been conducted at the same time each day; however, a 2-h difference in the start time of the daily treatment was accepted.

### Measured Parameters

All parameters were evaluated before the first treatment (M1) and after 3 (M2) and 6 months (M3) of treatment. The same double-blinded observer (NH) performed all measurements and was supervised by a specialist in canine sports medicine and rehabilitation (OH). The gait analysis was performed together with a double-blinded veterinary technician (AA) who had experience in performing objective gait analysis. The visual- and computer-based gait analyses were supplemented with pain scores and the measurement of the range of motion (ROM) of the affected joint.

#### Range of Motion

The range of motion was measured by placing the dogs in lateral recumbent position, and the goniometer was situated on the lateral part of the cubital joint overlying the palpable lateral epicondyle (13). The degree of the flexion and extension angles were measured out of a neutral position in order to calculate the range of motion (ROM). The elbow joint was passively conducted until the dogs exhibited signs of pain or when resistance became palpable.

#### Pain Score

Pain was evaluated using a simple, five-point ordinal scale (0–4) (14) that graded pain during palpation of the affected elbow.

#### Visual Gait Analysis

Dogs were instructed to trot on a short leash for 30 m on a flat ground in order to perform visual gait analysis. A subjective scale ranging from 0–5 was used to evaluate gait (14): 0 = no lameness observed, 1 = slight, intermittent lameness, 2 = obvious weight-bearing lameness, 3 = severe weight-bearing lameness, 4 = Intermittent non-weight-bearing lameness, and 5 = continuous non-weight bearing lameness. In some cases, a score of 0.5 was used for dogs that exhibited questionable lameness or if a consensus was not reached among the investigators and a mean score was calculated.

#### Objective Gait Analysis

Objective gait analysis was performed using a four-belt treadmill (Model 4060-08, Bertec Corporation, Columbus, Ohio, USA) with integrated force plates that measured the ground reaction forces in the x-, y-, and z-directions. Data were collected, processed, and exported to Microsoft Excel 2010 (Microsoft Excel 2010, Microsoft Corporation, Albuquerque, New Mexico, USA) using Vicon Nexus 1.8.5. software (Vicon Nexus 1.8.5., Vicon Motion Systems Ltd., Oxford, UK). After an acclimation period, the dogs were walked at a comfortable mean speed of 0.78 ± 0.14 m/s. In order to record valid trials, it was important that every paw strike was only detected on the appropriate force plate and that there were no oversteps (15). Furthermore, it was necessary to walk the dog as normal and straight as possible with the least amount of interference from the handling person (16, 17). Since velocity has a described effect of stance time and ground reaction forces, the same velocity was maintained for the control measurements (18, 19). The mean values of 10 steps were used to calculate the peak vertical force (PVFz) in the z-direction and the vertical impulse (IFz) (integral of the vertical force). For both parameters, the symmetry index (SI) was calculated using the following formula modified from Budsberg et al. (20):

$$\begin{array}{l} SI\;\left(\%\right)=100-\left[{\left(\text{Fa}/\text{Fc}\right)}^{*}100\right] \end{array}$$

*(SI* = *symmetry index of the corresponding parameter (PVFz, IFz); Fa* = *parameter of the affected thoracic limb; Fc* = *parameter of the contralateral thoracic limb)*.

A *SI* of zero signifies a perfect weight distribution of both limbs (20, 21).

#### Overall Treatment Score (OTE)

Similar to Mucha et al. (11), we calculated an overall treatment effectiveness (OTE) score at M2 and M3. The SIPVFz, the SIIFZ, the lameness, and the pain score were compared to their values in M1. Changes were defined as follow: +1, improvement; 0, no change; and −1, worsening. We used cut-off parameters of 3.7 and 3.5% for the PVFz and IFz, respectively (22). If the value of SI increased more than the cut-off parameter it was rated as worsening for this parameter, and a decreasing of the symmetry indices which amount was larger than the cut off parameter was rated as improvement. A decrease in the lameness score and/or pain score was rated as +1, no change was scored as 0, and an increase was rated as −1. If dogs required the additional use of medical treatments or physiotherapy, they received a score of −1. The OTE summarizes all scores and was only calculated for dogs with complete data.

### Statistical Analysis

For statistical data analysis the software SAS 9.4, using the “SAS Enterprise Guide” version 7.15 (SAS Institute Inc., Cary, NC, USA) was used.

The investigation on normal distribution of the parameters was done by shapiro wilks-test and visual assessment of qq-plots of the model residuals.

In case of normal distributed quantitative parameters as weight and age, a two sample *t*-test was used to analyze differences between the groups. In case of non-rejection of the normal distribution assumption, distribution free non-parametric methods were used. Wilcoxon Signed Rank-test was used to compare the PVFz, IFz, and ROM between the groups at M1, M2, and M3. Fisher's exact-test was used to calculate differences in the lameness- and pain scores at M1, M2, and M3 between groups. Fisher's exact-test was also used to evaluate differences in the additional use of NSAID's and physiotherapy between the TG and PG. The homogeneity of the sex distribution was tested by Fisher's exact-test.

For paired observations we conducted a Wilcoxon signed rank-test to analyze the differences between the OTE at M2 and M3 for each group. Fischer's exact-test was used to check the homogeneity distribution of OTE characteristics in the degrees of arthrosis, stratified by TG and PG.

A *p*-value of < 0.05 was considered statistically significant. The normal distributed data were presented as the mean ± standard deviation (SD). To present data that is not normally distributed the median and range is used.

## Results

### Study Population

Breeds were distributed as followed: Labrador Retriever (*n* = 17), Golden Retriever (*n* = 2), Australian Shepherd (*n* = 2), mixed breed (*n* = 2), Airdale Terrier (*n* = 1), Magyar Vizsla (*n* = 1), Old English Bulldog (*n* = 1), Pekinese (*n* = 1), Giant Schnautzer (*n* = 1). The weight and age of dogs were normally distributed, and there were no significant differences in weight (PG, 29.90 ± 6.84 kg; TG, 30.95 ± 8.74 kg; *p* = 0.73) and age (PG, 7.38 ± 3.68; TG, 6.48 ± 3.83; *p* = 0.53) between the PG and the TG. The gender distribution between the two groups is homogenous (*p* = 1.0).

Overall, two dogs dropped out of the TG: one after M1, and one after M2. One was lost to follow-up, and the other underwent surgery. One dog was unable to perform objective gait analysis at M2 due to fear of the treadmill. Four dogs dropped out of the PG. Two were lost to follow-up after M1, and two were lost to follow-up after M2. It was not suspected that these drop-outs were related to therapy.

Four animals per group received additional pain therapy for at least for weeks prior to M1. All dogs that received pain therapy at M1 continued to receive pain therapy at M2, and one dog per group started additional therapy at M2. At M3, all of the dogs continued receiving additional pain therapy, and one dog in the TG started additional pain therapy.

In both groups the median of the radiographic arthrosis score was 2 with a range from 1 to 3.

### Measured Parameters

**Figure 3:**
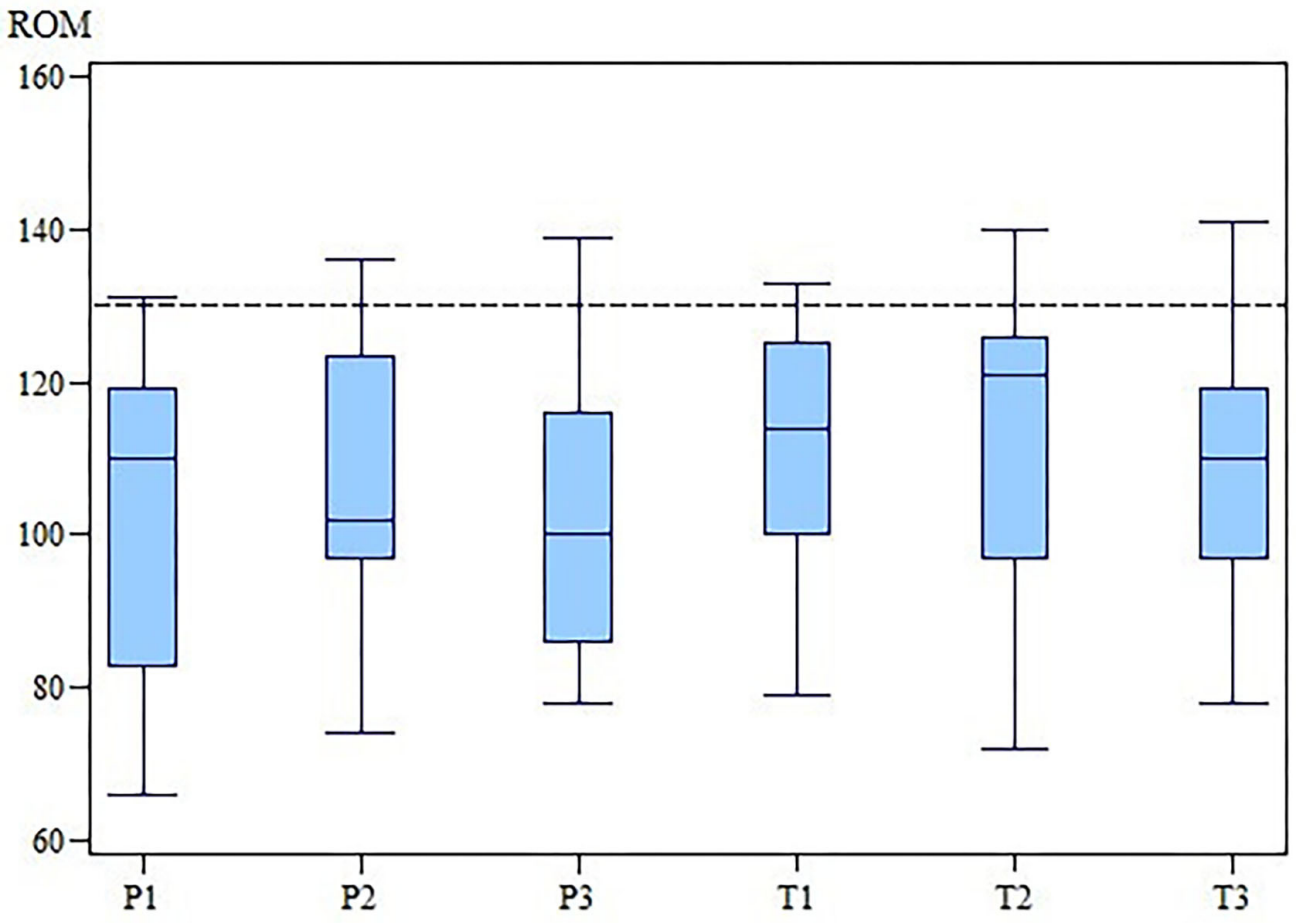
ROM of the affected elbow joint of the TG and the PG at M1, M2 and M3. While in the PG the ROM worsened, for TG there is an improvement at M2 and a drop down at M3. The differences between groups were not statistically significant. ROM, range of motion; TG, treatment group; PG, placebo group; M, measurement point. x-axis: Range of motion (ROM) in degree (°). y-axis: group (T for TG; P for PG) and measurement time (2 for M2; 3 for M3).

**Figure 4:**
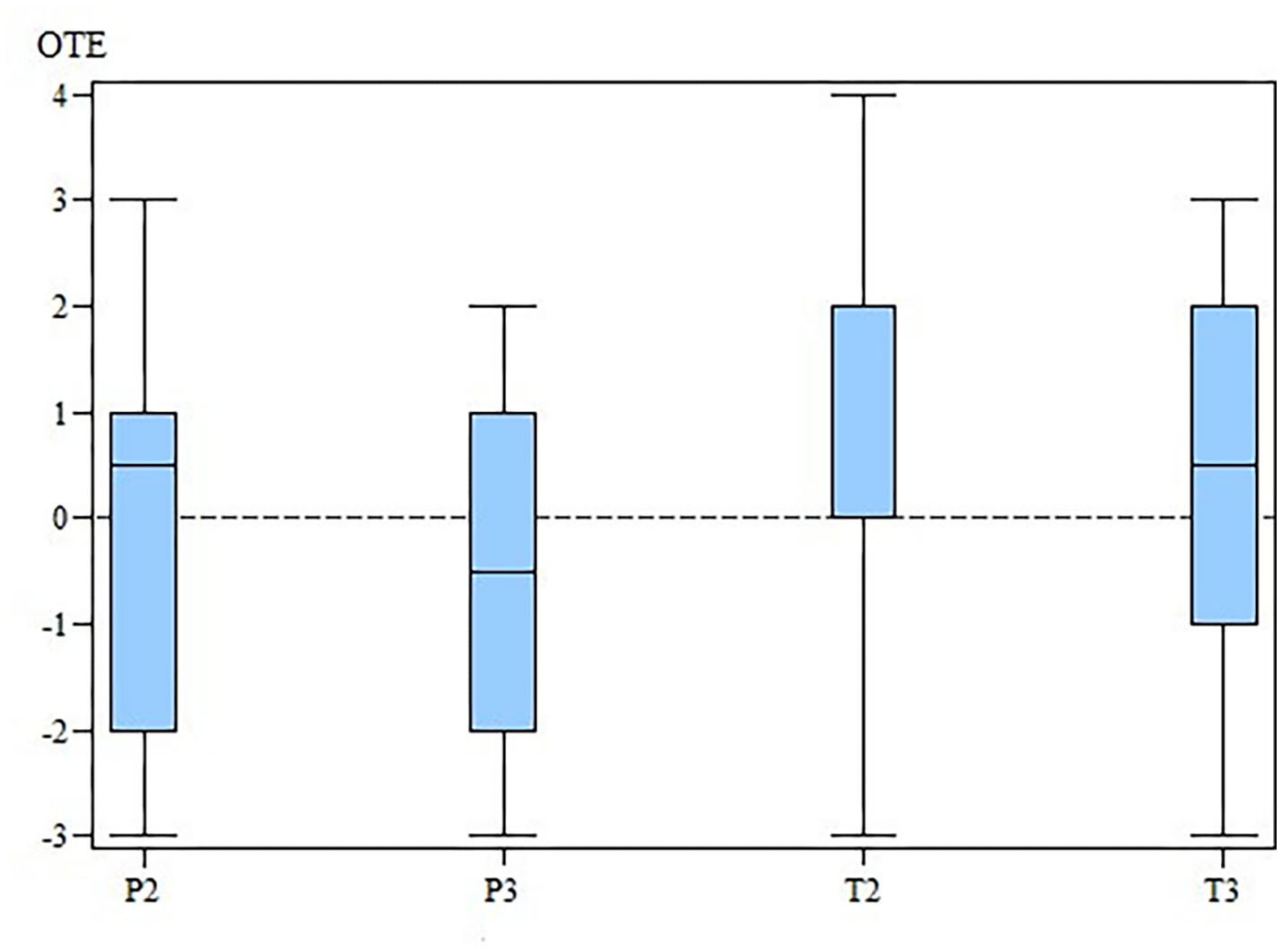
OTE for the TG and the PG at M2 and M3. OTE, overall treatment effectiveness; TG, treatment group; PG, placebo group; M, measurement point. x-axis: OTE, overall treatment effectiveness. y-axis: group (T for TG; P for PG) and measurement time (2 for M2; 3 for M3).

The changes in the ROM of the cubital joint and the OTE suggest that MBST® had a slightly positive effect in the OA treatment (Figures 3, 4). However, our results demonstrate that there is no statistically significant differences in SIPVFz, SIIFz, ROM, lameness, and pain scores between or within the groups.

At M1, the SIPVFz in the TG was 6.24% (range, 0.19–28.24%), and the SIPVFz in the PG was 7.99% (range, 0.96–18.61%). At M2, the SIPVFz in the TG was 5.22% (range, 1.29–15.14%), and the SIPVFz in the PG was 8.18% (range, 0.13–17.90%). At M3, the SIPVFz in the TG was 7.20% (range, 2.07–50.12%) and 9.45% (range, 0.73–26.12%) for the PG. At M2, the median SIPVFz was decreased in the TG and increased in the PG; however, this difference was not significant (M1, *p* = 0.33; M2, *p* = 0.22; M3, *p* = 0.55).

At M1 the SIIFz was 7.01% (range, 0.68–61.05%) in the TG and 12.59% (range, 1.39–27.25%) in the PG. At M2, the mean SIIFz was 7.50% (range, 2.12–15.97%) for the TG and 11.72% (range, 3.64–22.86%) in the PG. At M3 the mean SIIFz was 8.17% (range, 2.07–56.82%) in the TG and 8.94% (range, 0.20–32.57%) in the PG. There was no significant difference between groups (M1, *p* = 0.07; M2, *p* = 0.20; M3, *p* = 0.86).

The ROM at M1 was 109° (range, 79–133°) in the TG and 108.5° (range, 66–131°) in the PG. At M2, the ROM was 121° (range, 72–140°) in the TG and 102° (range, 74–136°) in the PG. At M3 the ROM was 110° (range, 78–141°) in the TG and 100° (range, 78–139°) in the PG. There was no significant difference in the ROM between the two groups at any time (M1, *p* = 0.33; M2, *p* = 0.68; M3, *p* = 0.40).

Additionally, there were no significant differences in the median lameness scores between the TG and PG at M1 [TG, 2 (range, 1–4); PG, 2 (range, 1–3); *p* = 0.56], M2 [TG, 1 (range, 0–3); PG, 1 (range, 0.5–3); *p* = 0.74], and M3 [TG, 1 (range, 0–3); PG, 1 (range, 0.5–3); *p* = 1.00]. Furthermore, there were no significant differences in the lameness scores within the two groups (TG, *p* = 0.48; PG, *p* = 0.9).

The pain scores showed no significant differences between groups at M1 [TG, 1 (range, 1–3); PG, 1.5 (range, 1–3); *p* = 0.76], M2 [TG, 1 (range, 0–3); PG, 2 (range, 0–3); *p* = 0.22], and M3 [TG, 1 (range, 0–2); PG, 1 (range, 0–2); *p* = 0.12]. Differences within groups were not significant (TG, *p* = 0.18; PG, *p* = 0.94).

At M2 the OTE values were 0.0 (range, −3–4) for TG and 0.5 (range, −3–3) for PG. At M3 the OTE was 0.5 (range, −3–3) for TG and −0.5 (range, −3–2) for PG. Finally, there were no significant differences in OTE scores (Tables 1–4) between the two groups at M2 (*p* = 0.78) and M3 (*p* = 0.41). The OTE characteristics were homogeneously distributed across the degrees of arthrosis at M2 (TG, *p* = 0.94; PG, *p* = 0.92) and M3 (TG, *p* = 0.83; PG, *p* = 0.57).

**Table 1:** Data of analyzed parameters to calculate the OTE for the TG at M2.

**Grade of** arthrosis^*^	**Lameness-score**	**SIPVFz**	**SIIFz**	**Pain-** **score**	**NSAID's**	**Physio-** **therapy**	**OTE**
3	0	0	−1	1	0	0	0
1	0	0	−1	0	−1	0	−2
2	0	0	0	0	0	0	0
2	0	−1	−1	−1	0	0	−3
1	1	*-*	*-*	0	0	0	
1	0	0	−1	1	0	0	0
2	1	*-*	*-*	1	0	0	
3	0	0	1	1	0	0	2
2	0	0	0	0	0	0	0
3	0	1	1	0	0	0	2
1	1	0	0	1	0	0	2
1	1	1	1	1	0	0	4
3	0	0	−1	1	0	0	0
2	*-*	*-*	*-*	*-*	*-*	*-*	

*The data were compared to their pre-treatment values (M1) and defined as follows: +1, improvement; 0, no change; and −1, worsening. Furthermore, the grade of the arthrosis in the affected joint was added to the table, regarding to the IEWG Scheme. OTE, overall treatment effectiveness; TG, treatment group; PG, placebo group; M, measurement point; SIPVFz, symmetry index of the peak vertical force; SIIFz, symmetry index of the vertical impulse; NSAID, non-steroidal anti-inflammatory drug*.

* *In the affected elbow joint according to the IEWG Scheme (23)*.

**Table 2:** Data of analyzed parameters to calculate the OTE for the TG at M3.

**Grade of** arthrosis^*^	**Lameness-score**	**SIPVFz**	**SIIFz**	**Pain-** **score**	**NSAID's**	**Physio-** **therapy**	**OTE**
3	1	0	0	1	0	0	2
1	−1	−1	−1	1	−1	0	−3
2	0	−1	0	0	0	0	−1
2	1	*-*	*-*	0	0	0	
1	1	−1	1	1	0	0	2
1	0	0	−1	1	0	0	0
2	1	1	1	0	0	0	3
3	0	−1	−1	1	0	0	−1
2	0	0	−1	1	0	0	0
3	0	0	−1	0	0	0	−1
1	1	0	0	0	0	0	1
1	*-*	*-*	*-*	*-*	*-*	*-*	
3	1	1	1	0	0	0	3
2	1	1	1	0	0	0	3

*The data were compared to their pre-treatment values (M1) and defined as follows: +1, improvement; 0, no change; and −1, worsening. Furthermore, the grade of the arthrosis in the affected joint was added to the table, regarding to the IEWG Scheme. OTE, overall treatment effectiveness; TG, treatment group; PG, placebo group; M, measurement point; SIPVFz, symmetry index of the peak vertical force; SIIFz, symmetry index of the vertical impulse; NSAID, non-steroidal anti-inflammatory drug*.

* *According to the IEWG Scheme (23)*.

**Table 3:** Data of analyzed parameters to calculate the OTE for the PG at M2.

**Grade of** arthrosis^*^	**Lameness-score**	**SIPVFz**	**SIIFz**	**Pain-** **score**	**NSAID's**	**Physio-** **therapy**	**OTE**
1	0	−1	−1	−1	0	0	−3
3	0	−1	−1	−1	0	0	−3
1	1	0	0	0	0	0	1
1	1	1	−1	0	0	0	1
2	0	−1	−1	0	0	0	−2
3	0	0	0	0	0	0	0
2	0	−1	0	0	−1	0	−2
3	1	−1	1	0	0	0	1
2	*-*	*-*	*-*	*-*	*-*	*-*	
1	0	0	1	0	0	0	1
2	1	0	0	−1	0	0	0
2	*-*	*-*	*-*	*-*	*-*	*-*	
1	1	1	1	0	0	0	3
3	1	1	1	0	0	0	3

*The data were compared to their pre-treatment values (M1) and defined as follows: +1, improvement; 0, no change; and −1, worsening. Furthermore, the grade of the arthrosis in the affected joint was added to the table, regarding to the IEWG Scheme. OTE, overall treatment effectiveness; TG, treatment group; PG, placebo group; M, measurement point; SIPVFz, symmetry index of the peak vertical force; SIIFz, symmetry index of the vertical impulse; NSAID, non-steroidal anti-inflammatory drug*.

* *According to the IEWG Scheme (23)*.

**Table 4:** Data of analyzed parameters to calculate the OTE for the PG at M3.

**Grade of** arthrosis^*^	**Lameness-score**	**SIPVFz**	**SIIFz**	**Pain-** **score**	**NSAID's**	**Physio-** **therapy**	**OTE**
1	0	−1	−1	−1	0	0	−3
3	0	0	1	0	0	0	1
1	0	−1	−1	0	0	0	−2
1	1	*-*	*-*	0	0	0	−1
2	0	−1	−1	0	0	0	−2
3	*-*	*-*	*-*	*-*	*-*	*-*	
2	1	0	1	0	−1	0	1
3	0	−1	1	0	−1	0	−1
2	*-*	*-*	*-*	*-*	*-*	*-*	
1	1	0	1	0	0	0	2
2	1	0	0	−1	0	0	0
2	*-*	*-*	*-*	*-*	*-*	*-*	
1	*-*	*-*	*-*	*-*	*-*	*-*	
3	0	1	1	0	0	0	2

*The data were compared to their pre-treatment values (M1) and defined as follows: +1, improvement; 0, no change; and −1, worsening. Furthermore, the grade of the arthrosis in the affected joint was added to the table, regarding to the IEWG Scheme. OTE, overall treatment effectiveness; TG, treatment group; PG, placebo group; M, measurement point; SIPVFz, symmetry index of the peak vertical force; SIIFz, symmetry index of the vertical impulse; NSAID, non-steroidal anti-inflammatory drug*.

* *According to the IEWG Scheme (23)*.

## Discussion

The purpose of this randomized, double-blind trial was to evaluate the clinical effects of MBST® on dogs with OA in the elbow joint. Previous human studies have reported positive effects of NMR therapy in patients with severe back pain, osteoporosis, and gonarthrosis (8–10). Furthermore, Mucha et al., (11) suspected that NMR therapy would be associated with positive effects in dogs with OA that affected different joints. In the current study, the SIPVFz and lameness score decreased in the TG after 3 months of treatment. However, we did not observe any significant differences in kinetic gait parameters, visual gait analysis, and ROM between the two groups at M1, M2, and M3. Below, we discuss reasons for the discrepancies between our results and those observed in human studies (8, 9, 24). First, we suggest that the heterogeneous nature of OA in humans is also observed in dogs (25, 26). Deveza and Loeser (27) previously defined human OA as a complex syndrome (26) with different characterized subgroups that are distinguished by prognosis, response to therapy, and the pathomechanism. Karsdal et al. (28) revealed that it was important to develop a method that differentiates between OA subtypes in order to provide optimal treatment for (human) patients and to develop therapies targeting specific subpopulations of OA. We suspect that the lack of significant effects between the TG and PG in the current study may be due to different OA subtypes. In most of the cases OA in the elbow joint of dogs is secondary, as a result of elbow dysplasia (29). Canine elbow dysplasia includes osteochondritis dissecans (OCD), medial coronoid process disease (MCPD), elbow incongruency (INC), and ununited anconeal process (UAP). It is suspected to be a multifactorial disease with secondary environmental influences (e.g., obesity, rapid growth, excessive work, and systemic inflammation) (30–33). Even if elbow dysplasia is the most likely cause for OA in the majority of the dogs participating in our study, other etiologies could not safely be excluded. A clinical tool to divide dogs with OA in different subtypes is currently not available but might be helpful, because under consideration of different subtypes of OA our selected group size might be too small or dominated by a subgroup which does not respond to therapy as well as others.

Another reason for these discrepancies may be the grade of the arthrosis and the relatively late stage of OA in our study population. According to the International Elbow Working Group scheme (23), a score of “2” is classified as moderate arthrosis. The median arthrosis score in our study was 2 with a range from 1 to 3 for the TG and PG. Taguchi et al. (34) found nutraceutical treatment efficacy on coxofemoral joint OA among low-grades but not high-grades. Farrell et al. (35) demonstrated that dogs with a higher grade of radiographic arthrosis had a significantly higher score of cartilage pathology compared to dogs with a low grade of radiographic arthrosis. Furthermore, an *in-vitro* study revealed that there is an NMR-induced increase in human chondrocyte and osteoblast proliferation (7). Although our study was not able to show a correlation between the grade of arthrosis and the response to NMR therapy, we suspect that NMR may have better effects in dogs with less damaged cartilage because more intact cells are available for stimulation in these animals. Although the exact mechanism of action is unknown, an effect at (cartilage) cell level is suspected. Oliva et al. (36) reported that NMR influenced circadian regulated and hypoxic pathways in zebrafish cells, and Gossan et al. (37) published a study that demonstrated that murine chondrocytes have an autonomous circadian clock that can be disrupted (e.g., during aging) and may increase susceptibility to joint disease. However, further studies are needed to understand the effects of NMR in mammalian chondrocyte cell lines. A better understanding of the cellular effects of NMR, pathogenesis of OA, and guidelines/methods used to categorize OA subtypes may help integrate MBST® into individualized treatment plans.

This study had multiple limitations. First, OA in the elbow joint frequently occurs in both elbows. Although we only included dogs that clearly demonstrated that lameness was associated with OA in one elbow joint, only three of the 28 dogs had unilateral changes, and the contralateral elbow showed borderline signs of elbow arthrosis in five dogs. Kinetic gait analysis only reflects limb function in comparison to the contralateral limb and not the joint specific function (38). To eliminate the adulteration of objective gait parameters by body weight, a symmetry index was used (20, 22), and we subsequently measured the ROM to evaluate functional changes after NMR therapy. Although there was no significant difference in the ROM between the two groups, the median ROM increased in the TG and the PG after 3 and 6 months of treatment. The goniometer is a reliable clinical tool, but in dogs with OA, the ROM may be voluntarily restricted due to expected pain and sensory-limited instead of mechanically (13). The type of end-feel was not further classified.

This study analyzed the effects of MBST® on the visual and objective gait analysis, a simple pain score, and the documentation of additional medical therapy or physiotherapy using an OTE score. At M2 and M3, the OTE values were not significantly different between the TG and PG. Nevertheless, the TG showed no change after 3 month and a positive overall improvement after 3 month, whereas the median OTE-value in the PG was positive after 3 month and negative after 6 month of treatment. The TG had the highest OTE-value after 6 months of treatment; however, Levers et al. (39) showed a period of worsening between 6 and 12 months of treatment. Therefore, more studies are needed to evaluate the long-term outcomes of NMR therapy.

The evaluation of chronic pain might be optimized in future studies by involving the pet owner and using a validated multifactorial clinical measurement tool, such as the Liverpool osteoarthritis score (LOAD) (40, 41). It should be mentioned that there are differences between gait analysis and subjective scores as Brown et al. (42) could show that owners focused differently on lameness than on behavioral changes of their dogs. A new approach that uses activity trackers might be helpful in the evaluation of the long-term response to NMR therapy (43).

Overall, a better understanding of the differences between human and canine OA may help to develop the MBST® protocol for canine OA.

## Conclusion

MBST® suggest a positive effect on the treatment of OA in dogs after 3 months but overall no significant effects could be shown. Nevertheless, further studies are needed to improve the understanding of the mechanism of NMR therapy and the pathophysiology of OA to provide optimal treatment options for individual patients. Additionally, due to the heterogenous nature of OA, a method that categorizes OA subtypes in dogs should be established.

## Data Availability Statement

All datasets generated for this study are included in the article/supplementary material.

## Ethics Statement

The protocol of this study was reviewed and approved by the Ethic Committee of the University of Veterinary Medicine Hannover (file number: TVO-2018-V-47). This study was carried out in accordance with the German animal welfare act within the law of animal welfare, Germany, and following the ethical guidelines of the University of Veterinary Medicine Hannover.

## Author Contributions

Clinical conception and design were done by NH, MF, and OH. Collection of data was done by NH, with assistance of DK in performing the objective gait analysis. NH performed data analysis, interpretation, and paper writing. OH supervised data collection and manuscript editing. All authors read and approved the final manuscript.

## Conflict of Interest

The authors declare that the research was conducted in the absence of any commercial or financial relationships that could be construed as a potential conflict of interest.
